# RPA-Mediated Recruitment of the E3 Ligase RFWD3 Is Vital for Interstrand Crosslink Repair and Human Health

**DOI:** 10.1016/j.molcel.2017.04.021

**Published:** 2017-06-01

**Authors:** Laura Feeney, Ivan M. Muñoz, Christophe Lachaud, Rachel Toth, Paul L. Appleton, Detlev Schindler, John Rouse

**Affiliations:** 1 MRC Protein Phosphorylation and Ubiquitylation Unit, School of Life Sciences, Sir James Black Centre, University of Dundee, Dundee DD1 5EH, Scotland; 2 Dundee Imaging Facility, School of Life Sciences, Sir James Black Centre, University of Dundee, Dundee DD1 5EH, Scotland; 3 Department of Human Genetics, University of Würzburg Biozentrum, 97074 Würzburg, Germany

**Keywords:** *RFWD3*, interstrand crosslink, Fanconi anemia, RPA, homologous recombination, ubiquitylation

## Abstract

Defects in the repair of DNA interstrand crosslinks (ICLs) are associated with the genome instability syndrome Fanconi anemia (FA). Here we report that cells with mutations in RFWD3, an E3 ubiquitin ligase that interacts with and ubiquitylates replication protein A (RPA), show profound defects in ICL repair. An amino acid substitution in the WD40 repeats of RFWD3 (I639K) found in a new FA subtype abolishes interaction of RFWD3 with RPA, thereby preventing RFWD3 recruitment to sites of ICL-induced replication fork stalling. Moreover, single point mutations in the RPA32 subunit of RPA that abolish interaction with RFWD3 also inhibit ICL repair, demonstrating that RPA-mediated RFWD3 recruitment to stalled replication forks is important for ICL repair. We also report that unloading of RPA from sites of ICL induction is perturbed in *RFWD3*-deficient cells. These data reveal important roles for RFWD3 localization in protecting genome stability and preserving human health.

## Highlights

• *RFWD3*-deficient human cells show profound defects in ICL repair
• RFWD3 regulates RPA dynamics to promote homologous recombination
• The FA-associated I639K mutation prevents RPA-dependent recruitment of RFWD3 to ICLs
• RPA32 mutations that abolish interaction with RFWD3 also inhibit ICL repair

## Graphical Abstract

**Figure:**
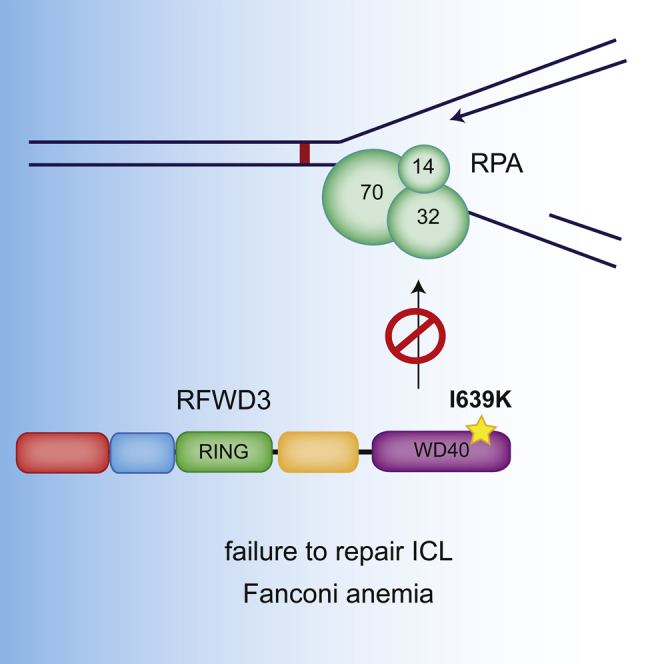

## Teaser

Feeney et al. show that the interaction of the E3 ubiquitin ligase RFWD3 with RPA plays a key role in the repair of DNA interstrand crosslinks, thereby preventing human disease. Loss of RFWD3 perturbs RPA dynamics at stalled replication forks and inhibits homologous recombination, a crucial step in ICL repair.

## Introduction

DNA interstrand crosslinks (ICLs) form when bifunctional agents covalently link the two strands in the DNA double helix. Although ICLs can be repaired in a replication-independent manner, the dominant mode of ICL repair is thought to occur in S phase, when replisomes collide with these lesions (Akkari et al., 2000, Taniguchi et al., 2002; Figure S1). ICL repair is a complex process requiring cooperation between several distinct DNA repair pathways (Kim and D’Andrea, 2012). The importance of efficient ICL repair is underscored by the finding that mutations in ICL repair are associated with Fanconi anemia (FA), a rare recessive disorder typified by developmental abnormalities, bone marrow failure, and increased incidence of cancers (Auerbach, 2009, Kim and D’Andrea, 2012). Twenty-one FA genes have been reported to date, and studying the products of these genes (the FANC proteins) has provided important insights into ICL repair.

The central components of the FA pathway, FANCD2 and its paralog FANCI, together form the “ID” complex (Garcia-Higuera et al., 2001, Smogorzewska et al., 2007). These proteins are mono-ubiquitylated at Lys561 and Lys523, respectively, both in S phase and in response to ICLs (Garcia-Higuera et al., 2001, Taniguchi et al., 2002). These reactions are catalyzed by the E3 ubiquitin ligase FANCL, a subunit of the FA core complex, in concert with the E2-conjugating enzyme FANCT and the FA-associated proteins FAAP100 and FAAP24 (Ciccia et al., 2007, Collis et al., 2008, Ling et al., 2007). Mono-ubiquitylation of FANCD2 is necessary for ICL repair (Kim and D’Andrea, 2012, Knipscheer et al., 2009); however, the underlying molecular mechanisms remain unclear. The FAN1 nuclease is recruited to stalled replisomes by ubiquitin-FANCD2, but this interaction is dispensable for ICL repair and is instead required for the protection of stalled forks in a general sense (Lachaud et al., 2016a, Lachaud et al., 2016b). FANCD2 ubiquitylation is known to promote lesion “unhooking,” involving nucleolytic incisions on either side of the ICL (Figure S1) that appear to require the XPF-ERCC1 nuclease (Knipscheer et al., 2009). After unhooking, the resulting gap is thought to be filled in by translesion synthesis. The unhooked lesion can subsequently be removed by excision repair, and the double-strand breaks (DSBs) generated by the unhooking step are ultimately repaired by homologous recombination (HR) (Figure S1).

Recently, it was reported that the RING finger-type E3 ubiquitin ligase RFWD3 affects HR at stalled replication forks (Elia et al., 2015). RFWD3 was already known to interact with the RPA32 subunit of the replication protein A (RPA) complex, a heterotrimeric complex essential for multiple aspects of DNA replication, repair, and recombination. Translocation of RFWD3 to sites of replication fork stalling appears to require interaction with RPA (Gong and Chen, 2011, Liu et al., 2011). More recently, it was shown that RFWD3 promotes RPA ubiquitylation in vivo in response to agents that cause fork stalling, such as hydroxyurea (HU), which depletes deoxyribonucleotide triphosphates (dNTPs). All three RPA subunits—RPA70, RPA32, and RPA14—appear to be ubiquitylated by RFWD3, but the functional effect of RFWD3-mediated ubiquitylation on RPA is not yet clear. Cells depleted of RFWD3 show defects in restart of forks stalled after exposure of cells to HU and also show defects in HR at a protein-DNA roadblock that causes fork stalling (Elia et al., 2015). However, it remains to be seen whether RPA ubiquitylation is relevant to the fork-protective functions of RFWD3 or, indeed, whether the E3 activity of RFWD3 is required. Attenuation of the RPA-RFWD3 interaction through deletion of a small region in the C terminus of RPA32 not only abolishes RPA ubiquitylation but also reduces the efficiency of HR at a protein-DNA roadblock. However, mutating the ubiquitylated residues K37 and K38 in RPA32 inhibited RPA32 ubiquitylation without affecting HR (Elia et al., 2015). Therefore, it is not yet clear whether RPA ubiquitylation is relevant for DNA repair or whether, instead, RPA simply recruits RFWD3 to stalled forks, where it ubiquitylates other substrates to control genome stability.

Here we show that interfering with RFWD3 function causes key hallmarks of defective ICL repair. An *RFWD3* mutation found in a new FA subtype inhibits ICL repair by abolishing the interaction of RFWD3 with RPA. Moreover, RFWD3 controls RPA dynamics, and point mutations in RPA that abolish its interaction with RFWD3 attenuate ICL repair. These data reveal that the interaction of RFWD3 with RPA is vital for ICL repair and for preserving human health.

## Results

### Generating *RFWD3*-Deficient Human Cells

Recently, mutations in the gene encoding the E3 ubiquitin ligase RFWD3 were found in a new FA subtype, suggesting a role for RFWD3 in ICL repair (D.S., unpublished data). To test this possibility, we used CRISPR/Cas9-mediated genome editing to disrupt the *RFWD3* gene in HeLa cells. Although we succeeded in knocking out other genes, including *ERCC1* (Figure S2A), we failed to obtain clones that completely lacked RFWD3 protein even after using a range of guide RNAs (gRNAs). Similar results were obtained in U2OS cells (data not shown), suggesting that RFWD3 might be essential for cell viability. However, analysis of genomic DNA from a range of HeLa cell clones obtained using a gRNA targeting exon 4 revealed a high frequency of homozygous alterations at the *RFWD3* locus (data not shown). We reasoned that these clones might harbor hypomorphic *RFWD3* mutations that could affect DNA repair. We therefore screened clones for the ability to survive exposure to a single dose of the ICL-inducing drug mitomycin-C (MMC) using the low-sensitivity, high-throughput MTS assay. As shown in Figure S2B, several clones obtained after targeting *RFWD3* exon 4 showed reduced ability to survive MMC exposure compared with control HeLa cells. RT-PCR analysis of mRNA isolated from clone 7, for example, revealed compound heterozygosity at the *RFWD3* locus. One allele harbors a deletion of 13 bases within exon 4 and an insertion of four random bases within the gRNA-binding sequence, which together resulted in replacement of amino acids 254–258 (IDGGK) in the linker domain with two random amino acids (NW), after which the RFWD3 sequence is normal right to the end of the reading frame (Figures S2C–S2E). The second allele harbors a deletion in exon 4, which changes the frame and introduces a premature stop codon, thereby truncating the protein from Glu240, with the introduction of six extra amino acids (Figures S2F–S2H). Western blotting of cell extracts of clone 7 revealed an RFWD3 species of similar molecular weight as wild-type (WT) RFWD3 (Figure S2A), which we presume to be the product of the first allele mentioned above (Figures S2C–S2E). We refer to the *RFWD3*-hypomorphic HeLa cells as *RFWD3*^Δ/Δ^ cells hereafter.

### *RFWD3*^Δ/Δ^ Cells Show Hallmarks of Defective ICL Repair

**Figure 1:**
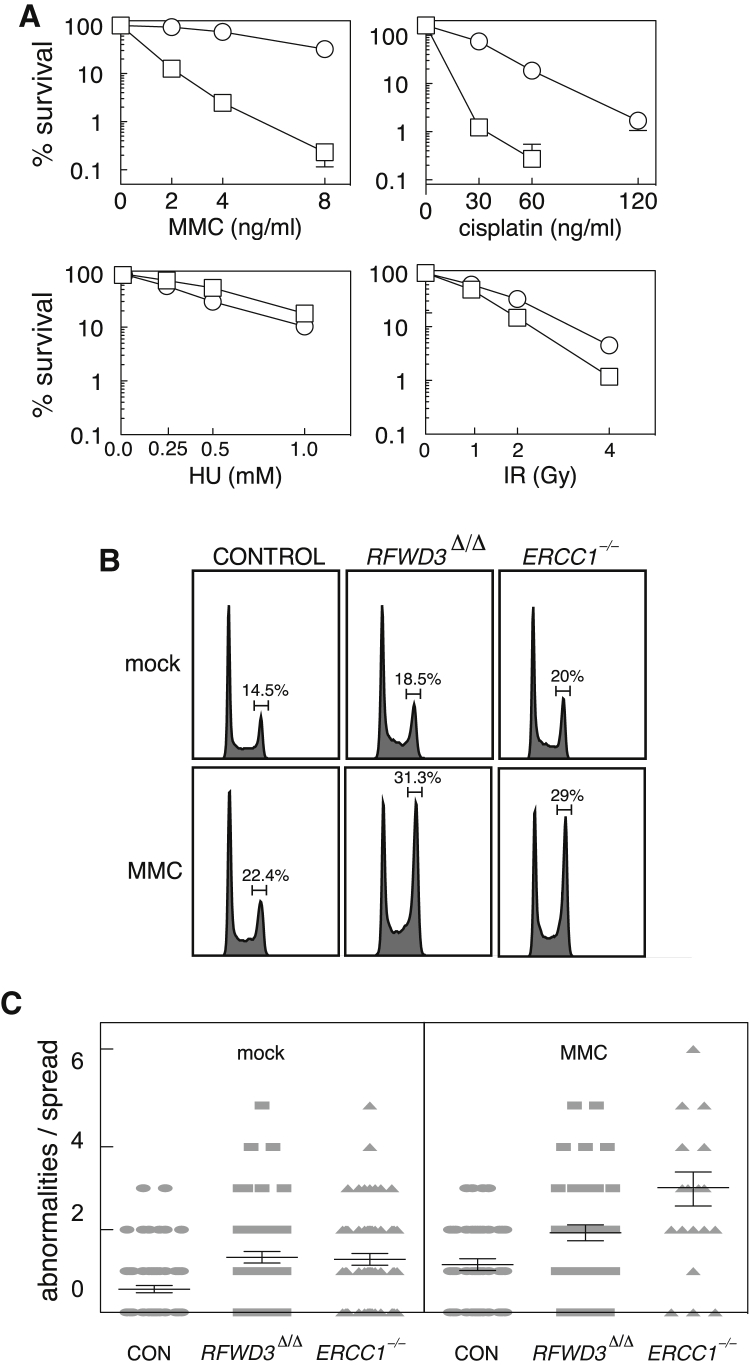
*RFWD3*-Deficient Cells Show Hallmarks of Defective ICL Repair (A) Clonogenic survival analysis of control and *RFWD3*^Δ/Δ^ HeLa cells exposed to the indicated genotoxins. For each cell type, the viability of untreated cells is defined as 100%. Data are represented as mean ± SEM; n = 3. Control cells are parental cells that were taken through genome editing protocols but are WT for RFWD3. Circles, control; squares, *RFWD3*^Δ/Δ^. (B) Cells were left untreated (mock) or treated with MMC for 24 hr before ethanol fixation, propidium iodide staining, and fluorescence-activated cell sorting (FACS) analysis. The proportion of cells in G_2_ phase is indicated in each case. (C) Metaphase spreads were prepared from cells of the indicated genotypes that were left untreated or treated with MMC for 24 hr. The number of chromosome breaks and radial chromosomes per metaphase spread was quantified. Data are represented as mean ± SEM.

To more accurately measure the sensitivity of *RFWD3*^Δ/Δ^ cells to DNA crosslinking agents, we decided to carry out clonogenic survival assays. As shown in Figure 1A, *RFWD3*^Δ/Δ^ cells are profoundly hypersensitive to agents that induce ICLs, such as MMC and cisplatin. The extreme sensitivity of *RFWD3*^Δ/Δ^ cells to these crosslinking agents is comparable with HeLa cells lacking *ERCC1*, a gene that encodes a subunit of the FA protein complex ERCC1-XPF (Bogliolo et al., 2013; Figure S3A). In contrast, *RFWD3*^Δ/Δ^ cells show no hypersensitivity to HU, which stalls replication forks by depleting dNTPs, and are only weakly sensitive to ionizing radiation (IR), which induces single- and double-strand breaks (Figure 1A). The narrow spectrum of genotoxins (ICL-inducing agents) to which *RFWD3*^Δ/Δ^ cells show sensitivity argues strongly that ICL repair is a major function of RFWD3. We therefore tested *RFWD3*^Δ/Δ^ cells for other hallmarks of defective ICL repair. Exposure to a low dose of MMC caused a high proportion of *RFWD3*^Δ/Δ^ cells, but not control cells, to accumulate in G_2_ phase. This pronounced G_2_ arrest is a hallmark of FA cells, and, indeed, the strength of G_2_ arrest seen in *RFWD3*^Δ/Δ^ cells is similar to that in *ERCC1*^−/−^ cells (Figure 1B). We also noticed that *RFWD3*^Δ/Δ^ cells showed an increased incidence of spontaneous chromosome abnormalities, defined by chromatid breaks and radial chromosome structures (Figure 1C). The frequency of abnormalities increased after exposure to MMC to a level higher than observed in control cells (Figure 1C). Again, the increased frequency of chromosome abnormalities in *RFWD3*^Δ/Δ^ cells was comparable with *ERCC1*^−/−^ cells.

We also carried out a crude epistasis-type experiment by depleting FANCD2 from *RFWD3*^Δ/Δ^ cells. Cells defective in both RFWD3 and FANCD2 were not more sensitive to MMC than *RFWD3*^Δ/Δ^ cells (Figures S3B and S3C), which strongly suggests that RFWD3 is part of the FA network, consistent with the identification of RFWD3 as a bona fide FANC protein. Furthermore, the ubiquitylation of FANCD2 induced by exposure of cells to MMC occurs normally in *RFWD3*^Δ/Δ^ cells (Figure S3D). Taken together, these data reveal that RFWD3 is important for the repair of ICLs and appears to act downstream of FANCD2 in the FA pathway.

### The FA-Derived I639K Mutation Abolishes RFWD3 Interaction with RPA

**Figure 2:**
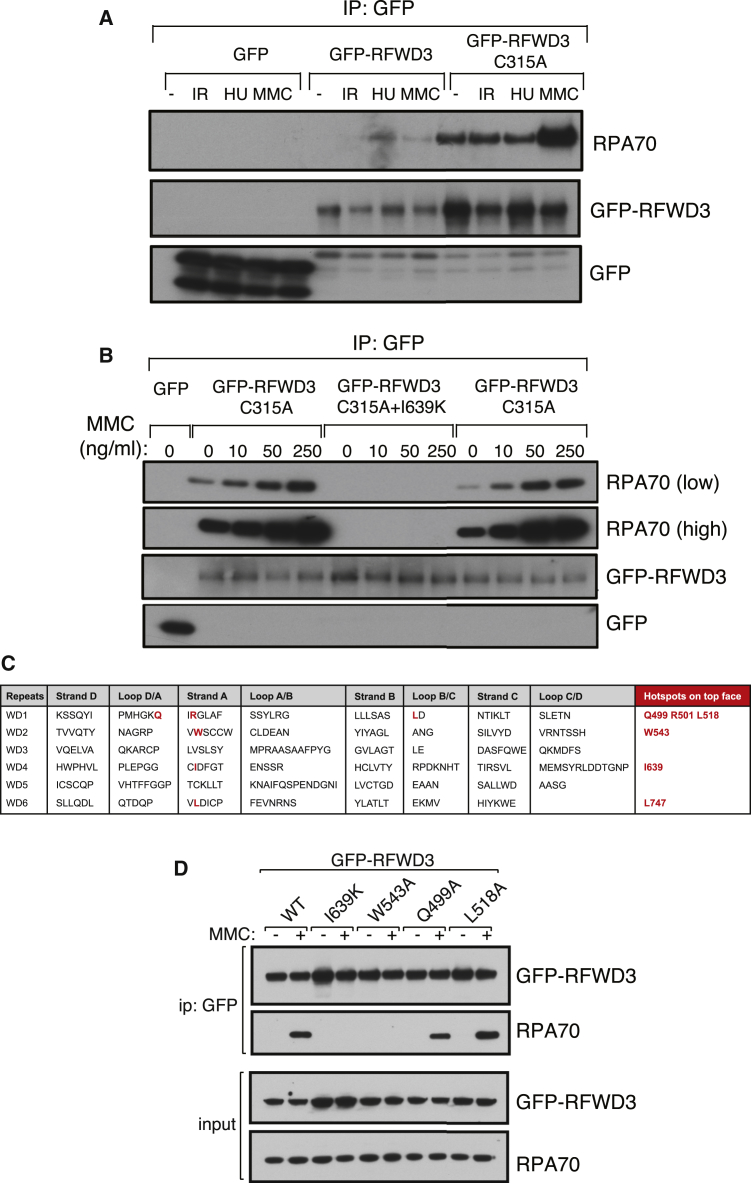
The FA-Derived RFWD3 I639K Mutant Cannot Interact with RPA (A) U2OS cells stably expressing GFP-RFWD3 (WT or a catalytically inactive C315A mutant) or GFP only were exposed to HU (2 mM) or MMC (50 ng/mL) for 16 hr and then lysed. Anti-GFP immunoprecipitates were probed with the indicated antibodies. In the case of IR, cells were exposed to 10 Gy and left to recover for 1 hr before lysis. (B) U2OS cells stably expressing GFP-RFWD3 (C315A or C315A I639K) or GFP only were exposed to the indicated concentrations of MMC for 16 hr and then lysed and analyzed as in (A). (C) The human RFWD3 amino acid sequence was entered into the WD40 repeat prediction algorithm at the WDSPdb database. The table predicts six WD40 repeats (WD 1–6). For each repeat, the sequence of the four anti-parallel β strands typical of WD40 repeats is given, as well as the sequence of loops connecting these β strands. In red are shown predicted surface hotspots that, in other WD40 domain proteins, mediate key protein-protein interactions. (D) Same as (B), except that U2OS cells expressing GFP-RFWD3 C315A or GFP-RFWD3 C315A bearing mutations in the predicted WD40 hotspots described in (C) were treated with a single dose of MMC for 16 hr before analysis.

As mentioned above, a new FA subtype has recently been identified that is associated with mutations in *RFWD3,* termed hereafter FA-RFWD3 (D.S., unpublished data). So far, only one patient has been identified, showing compound heterozygosity at the *RFWD3* locus (Figure S4). In one allele, a frameshift results in the introduction of a premature stop codon, which should truncate the protein product at amino acid Leu69. In contrast, the second allele harbors a single point mutation, I639K, located within the WD40 domain at the C terminus of RFWD3 (Figure S4). It is likely that the RFWD3-I639K point mutant is the only form of RFWD3 expressed in FA-RFWD3 cells. In light of this, we wanted to investigate the effect of this mutation on the ICL repair functions of RFWD3. We reasoned that the I639K mutation may disrupt the interaction of RFWD3 with RPA, and we set out to test this possibility. RPA was detected in anti-GFP immunoprecipitates from cells stably expressing GFP-tagged RFWD3 (Figure 2A), although the signal was weak and varied widely between experiments. We found that introducing a C315A mutation in the active site of RFWD3, which renders the recombinant protein inactive in vitro (Figure S5), stabilized RFWD3, resulting in increased expression, and made the interaction with RPA easier to detect (Figure 2A). Exposure of cells to MMC resulted in a dose-dependent increase in the amount of RPA in RFWD3 precipitates (Figure 2B); we observed a similar increase in several independent experiments, but the fold increase varied between experiments for reasons that are unclear (data not shown). Remarkably, introducing the FA-associated I639K mutation completely abolished the co-immunoprecipitation of RPA with GFP-RFWD3 C315A without affecting RFWD3 C315A expression levels (Figure 2B).

Ile639 is located within the WD40 domain of RFWD3, toward the C terminus (Figure S4). WD40 domains are known to be involved in mediating protein-protein interactions and are generally composed of six to eight structurally conserved WD40 repeats. Each of these repeats contains four anti-parallel β strands that fold into a conserved β-propeller structure, exposing three types of surface—top, bottom, and side (Stirnimann et al., 2010). Certain “hotspot” residues on these surfaces engage in protein-protein interactions that play important roles in various cellular functions (Wu et al., 2003). Recently, a database of WD40 proteins was reported (WDSPdb) that uses an algorithm to identify WD40 repeats in query proteins, predict secondary structure, and pinpoint surface hotspot residues (Wang et al., 2015b). This algorithm revealed six WD40 repeats in RFWD3 and predicted six top-face hotspots likely to engage in protein-protein interactions. Interestingly, this list included I639, located in the fourth WD40 repeat (Figure 2C). We therefore decided to test whether, like I639, mutation of the other predicted hotspots also affected the interaction of RFWD3 with RPA. As shown in Figure 2D, mutation of W543, but not Q499 or L518, to alanine completely abolished the interaction of GFP-tagged RFWD3 with RPA. These data show that the FA-associated I639K mutation prevents the binding of RFWD3 to RPA and that the surface-exposed residue W543 is also critical for this interaction.

### The I639K Mutation Abolishes RFWD3 Recruitment to DNA Damage Sites

**Figure 3:**
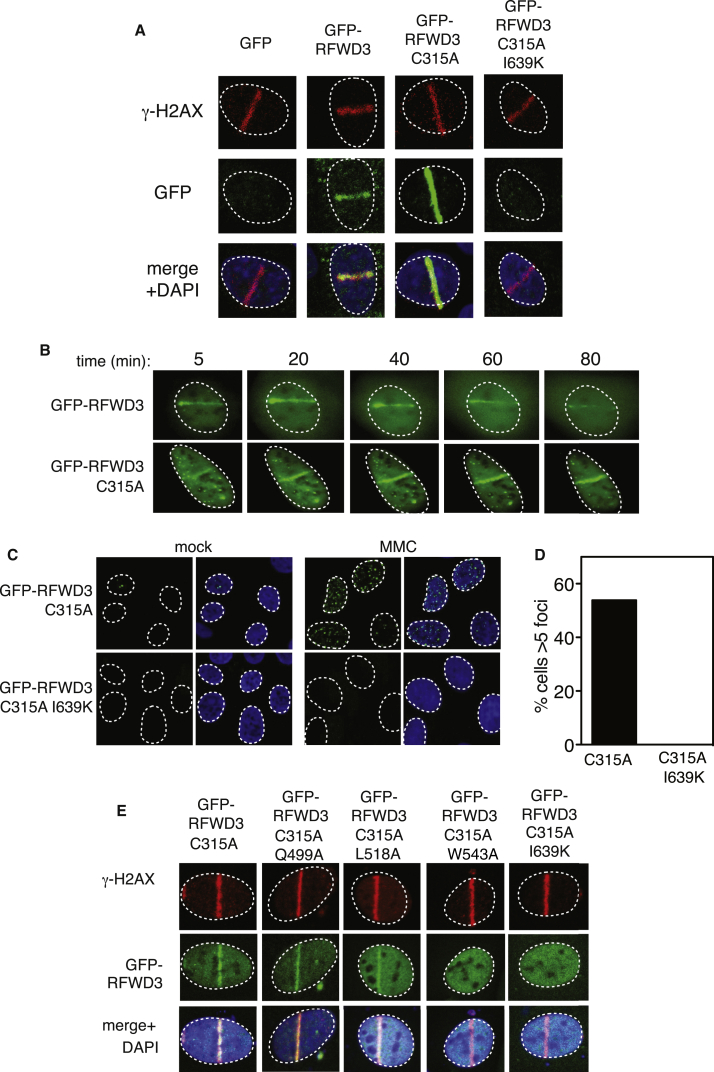
The RFWD3 I639K Mutant Is Not Recruited to DNA Damage Sites (A) U2OS cells stably expressing GFP, GFP-RFWD3, GFP-RFWD3 C315A, or GFP-RFWD3 C315A I639K were incubated with trimethyl-psoralen and subjected to sub-nuclear micro-irradiation using a 355-nm UV laser. Cells were fixed and subjected to indirect immunofluorescence analysis with antibodies against GFP or γ-H2AX. (B) Time-lapse live imaging of U2OS cells stably expressing GFP-RFWD3 (WT or C315A) incubated with trimethyl-psoralen and subjected to sub-nuclear micro-irradiation using a 355-nm UV laser. The time after micro-irradiation is indicated. (C) U2OS cells stably expressing GFP-RFWD3 C315A or GFP-RFWD3 C315A I639K were left untreated (mock) or exposed to MMC for 16 hr. Cells were fixed and subjected to indirect immunofluorescence analysis with antibodies against GFP or γ-H2AX. (D) Quantitation of the proportion of cells in (C) with more than five RFWD3 foci. (E) GFP-RFWD3 C315A or GFP-RFWD3 C315A bearing the WD40 repeat mutations indicated were analyzed as in (A).

Because interaction with RPA has been implicated in the recruitment of RFWD3 to sites of HU-induced fork stalling (Gong and Chen, 2011, Liu et al., 2011), we reasoned that the FA-associated I639K mutation might have an effect on RFWD3 localization to ICLs. To investigate this possibility, we utilized ultraviolet (UV) laser-activated psoralen conjugates to generate ICLs along a track in the nucleus and then visualized recruitment of GFP-tagged RFWD3 to these tracks by indirect immunofluorescence. As shown in Figure 3A, RFWD3 with a GFP tag at the N terminus, but not GFP alone, translocates to tracks of laser micro-irradiation and co-localizes with the phosphorylated form of histone variant H2AX (γ-H2AX). The intensity of the stripes formed by the C315A catalytically inactive RFWD3 mutant was noticeably higher than that of WT RFWD3 (Figure 3A). Live imaging analysis revealed that GFP-RFWD3 is recruited to ICLs within 5 min of ICL induction, reaching maximum intensity at around 20 min (Figure 3B). By 80 min, the GFP-RFWD3 stripe had almost disappeared. The C315A mutant was recruited with comparable kinetics, although, again, the stripe intensity was higher than that of the WT. Intriguingly, however, the C315A mutant persisted at sites of ICL induction and appeared to continue to increase in intensity even at the 80-min time point (Figure 3B). These data suggest that RFWD3 activity is necessary for its own removal from DNA damage sites but not for its recruitment. Strikingly, introducing the I639K mutation prevented localization of GFP-RFWD3 C315A to tracks of ICL induction (Figure 3A). To confirm these results, we also examined RFWD3 localization to sub-nuclear foci induced by exposure to MMC. As shown in Figure S6, GFP-RFWD3 C315A forms subnuclear foci in response to MMC treatment, and these foci co-localize with RPA. However, the I639K mutation completely abolishes formation of MMC-induced RFWD3 foci (Figures 3C and 3D).

We next tested the effect of mutating the WD40 surface hotspot residues described above (Figure 2C) on RFWD3 localization. As shown in Figure 3E, mutating Q499 or L518, which did not affect interaction of RFWD3 with RPA, had little effect on recruitment of GFP-RFWD3 to sites of ICL induction. However, mutating W543, which prevents interaction with RPA, abolishes recruitment of GFP-RFWD3 (Figure 3E), similar to the I639K mutation. Therefore, only WD40 mutations that abolish RPA binding prevent recruitment of RFWD3 to sites of ICL induction.

### The I639K Mutation Abolishes ATR-Catalyzed RFWD3 Phosphorylation

**Figure 4:**
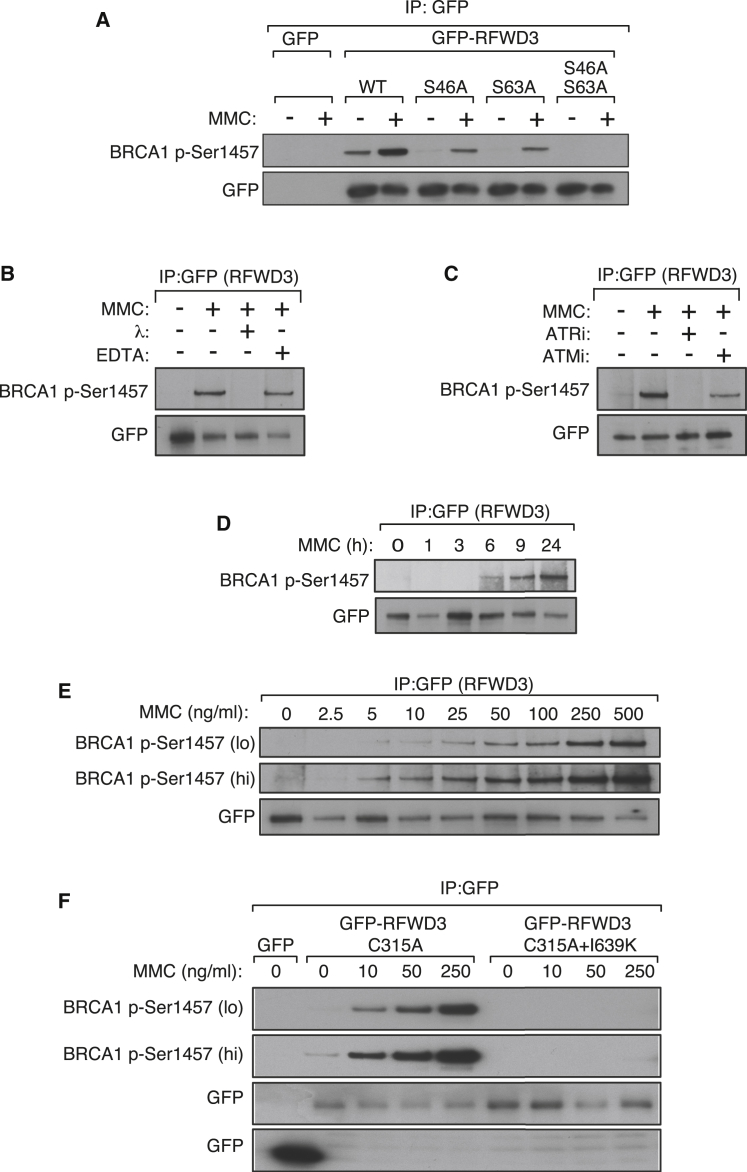
Defective Phosphorylation of RFWD3 I639K (A) U2OS cells stably expressing GFP only, GFP-RFWD3 WT, or GFP-RFWD3 bearing the S46A or S63A mutations singly and in combination were exposed to MMC for 16 hr. Anti-GFP precipitates were probed with the antibodies indicated. (B) GFP-RFWD3 was immunoprecipitated from extracts of U2OS cells exposed or not to MMC for 16 hr. Precipitates were incubated or not with λ-phosphatase in the presence or absence of EDTA before western blotting with the indicated antibodies. (C) U2OS cells stably expressing GFP-RFWD3 were preincubated with or without the ATR inhibitor ETP-46464 (5 μM) or the ATM inhibitor KU-55933 (5 μM) and then analyzed as in (A). (D and E) U2OS cells stably expressing GFP-RFWD3 were exposed to MMC at the indicated doses for 16 hr (D) or with MMC (100 ng/mL) for the indicated times (E) and analyzed as in (A). (F) U2OS FRT cells stably expressing GFP-RFWD3 (C315A or C315A + I639K) were treated with the indicated doses of MMC for 16 hr. Cells were lysed, and anti-GFP precipitates were probed with the indicated antibodies.

It has been reported previously that RFWD3 is phosphorylated by the ataxia telangiectasia mutated (ATM) and ataxia telangiectasia mutated-related (ATR) kinases following ionizing radiation (Fu et al., 2010, Matsuoka et al., 2007, Mu et al., 2007). An antibody against BRCA1 phospho-S1457 was reported to recognize phospho-RFWD3 after IR (Fu et al., 2010), and we found that GFP-RFWD3, but not GFP, cross-reacts with these antibodies after exposure of cells to MMC (Figure 4A). The residues flanking S46 and S63 of RFWD3 resemble those around BRCA1 S1457 (Fu et al., 2010), and, accordingly, mutating both of these residues together completely abolished the cross-reactivity of GFP-RFWD3 with the BRCA1 phospho-S1457 antibody after MMC treatment (Figure 4A). Phosphatase treatment of GFP-RFWD3 immunoprecipitates also abolished cross-reactivity with BRCA1 phospho-S1457 antibodies (Figure 4B). Furthermore, pre-incubation of cells with the specific ATR inhibitor ETP-46464 (Toledo et al., 2011), but not the ATM inhibitor KU-55933 (Hickson et al., 2004), blocked RFWD3 phosphorylation after MMC, suggesting that this phosphorylation is predominantly mediated by the ATR kinase (Figure 4C). As shown in Figures 4D and 4E, MMC-induced RFWD3 phosphorylation is both dose and time dependent. RFWD3 phosphorylation is not observed until around 6 hr after exposure of cells to MMC, with the phospho-specific signal increasing up to 24 hr. This lag in RFWD3 phosphorylation suggests that entry to S-phase is required, which is consistent with the requirement for ATR. As shown in Figure 4F, introducing the FA-associated I639K mutation completely abolished phosphorylation of GFP-RFWD3 C315A.

### Altered RPA Dynamics at ICLs in *RFWD3*-Defective Cells

**Figure 5:**
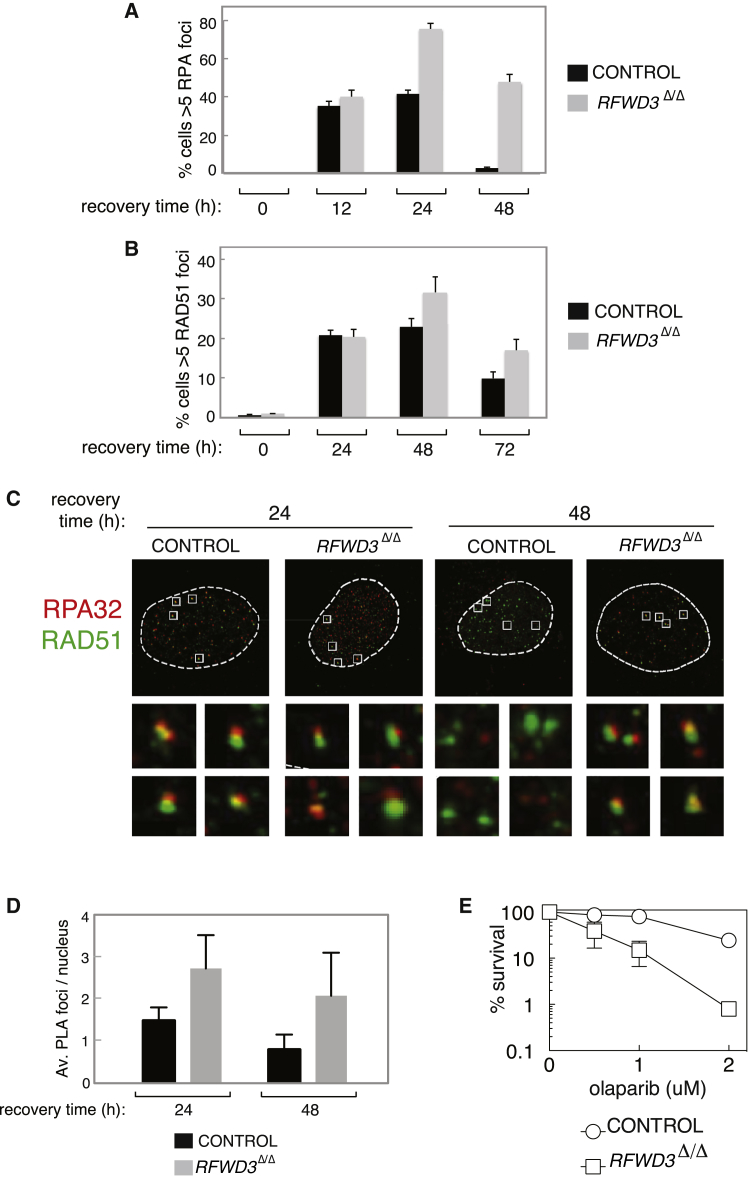
Altered RPA Dynamics at ICLs in *RFWD3*-Deficient Cells (A and B) Control or *RFWD3*^Δ/Δ^ HeLa cells were exposed to MMC for 2 hr and then washed free of drug and allowed to recover for the indicated times. Cells were pre-extracted, fixed, and subjected to immunofluorescence analysis to detect the proportion of cells with more than five RPA32 foci (A) or RAD51 foci (B). Control cells were parental cells that were taken through genome editing protocols but were WT for RFWD3. Data are represented as mean ± SEM, n = 3. (C) Control or *RFWD3*^Δ/Δ^ HeLa cells were exposed to MMC for 2 hr and then washed free of drug and allowed to recover for the indicated times. Cells were then treated, fixed, and subjected to immunofluorescence as in (A) (top). Individual nuclei were then imaged at high resolution (63× oil immersion lens, pinhole 0.5 Airy unit [AU]) to examine the structure of RPA32 and RAD51 foci (bottom). (D) Control or *RFWD3*^Δ/Δ^ HeLa cells were exposed to MMC for 2 hr and then washed free of drug and allowed to recover for the indicated times. Cells were then incubated with RPA32 and RAD51 primary antibodies conjugated to different oligonucleotides. Ligation and amplification with fluorescent dNTPs were then carried out, and the number of fluorescent foci per nucleus was quantified for each sample. Data are represented as mean ± SEM; n = 3. (E) Clonogenic survival analysis of control and *RFWD3*^Δ/Δ^ HeLa cells subjected to chronic treatment with the indicated doses of olaparib. For each cell type, the viability of untreated cells is defined as 100%. Data are represented as mean ± SEM; n = 3.

We next set out to test the effect of RFWD3 on RPA dynamics at sites of ICL induction. To this end, the proportion of cells with more than five RPA32 foci after exposure of cells to a transient pulse of MMC was quantitated. As shown in Figure 5A, the proportion of control cells with more than five RPA32 foci rose to reach a maximum of around 40% after 12–24 hr, declining to near-basal levels by 48 hr. The proportion of *RFWD3*^Δ/Δ^ cells with more than five RPA32 foci rose to almost 80% at the 24-hr time point and started to decline thereafter (Figure 5A). However, at 48 hr, when hardly any control cells had RPA foci, around 45% of *RFWD3*^Δ/Δ^ cells still had more than five foci. A similar difference between control and *RFWD3*^Δ/Δ^ cells was observed when the number of RPA foci per cell was quantitated (Figure S7A).

During HR, RPA should be displaced from single-stranded DNA (ssDNA) to allow formation of the RAD51 nucleofilament, and we wondered whether the persistence of RPA at sites of ICL induction in *RFWD3*-defective cells might prevent the loading of RAD51. We investigated this possibility by analyzing the appearance and disappearance of RAD51 foci from sites of ICL induction in the same manner as described above for RPA32. In control cells, RAD51 loaded onto sites of ICL induction, as assessed by quantitating the proportion of cells with more than five foci, reaching a maximum and plateauing by 24 hr (Figure 5B). By 72 hr, the foci had declined substantially. Intriguingly, RAD51 appeared to load normally in *RFWD3*^Δ/Δ^ cells, and, if anything, a slightly higher proportion of *RFWD3*^Δ/Δ^ cells had more than five RAD51 foci compared with control cells. In both cell lines, RAD51 foci had declined in number by 72 hr, although the proportion of *RFWD3*^Δ/Δ^ cells with RAD51 foci was still notably higher than WT. (Figures 5B and S7B). It is interesting to note that at the 48 hr post-recovery time point, *RFWD3*^Δ/Δ^ cells had close to normal levels of RAD51 foci despite failure of RPA to dissociate.

We next employed high-resolution microscopy to investigate the co-localization of RPA and RAD51 at times during recovery when RPA foci were found to persist in *RFWD3*^Δ/Δ^ cells. Twenty-four hours after recovery from a pulse of MMC, around 20% of RAD51 foci in both *RFWD3*-deficient and control cells also contained RPA (Figure 5C, top). Broadly speaking, two notable patterns of RAD51 and RPA localization were evident. The first pattern involved RAD51 and RPA occupying opposite ends of the same subnuclear focus, with co-localization (defined by signal overlap) restricted to a central zone. The second pattern involved RPA staining along the whole focus, with RAD51 co-localization occurring in a central zone (Figure 5C, bottom). At the 24-hr recovery time point, *RFWD3*^Δ/Δ^ cells were indistinguishable from control cells in the distribution of the two broad patterns of localization of RPA and RAD51. By the 48-hr recovery time point, almost all RPA foci had disappeared from control cells, and, consequently, the RAD51 foci did not contain RPA (Figure 5C, top). However, in *RFWD3*^Δ/Δ^ cells where RPA foci persisted, around 30% of RAD51 foci still contained RPA (Figure 5C). The patterns of RAD51 and RPA localization under these conditions were indistinguishable from control cells at the earlier time point (Figure 5C, bottom).

To validate these results, we also applied in situ proximity ligation assay (PLA) technology. In this experiment, the RAD51 and RPA primary antibodies are conjugated with two different oligonucleotides. When in close proximity, ligation of the oligonucleotide moieties becomes possible, creating a DNA sequence that can be PCR-amplified. In the presence of fluorescent dNTPs, a fluorescent signal is generated. As shown in Figures 5D and S7C, a small number of distinct foci could be seen in both control and *RFWD3*-deficient cells after 24 hr of recovery from an MMC pulse. The number of foci visible in control cells significantly decreased after 48 hr of recovery; however, the number of PLA foci in *RFWD3*-deficient cells remained notably higher.

Taken together, these data show that RPA persists in *RFWD3*-defective cells, resulting in the presence of RPA in a subset of RAD51 foci at time points where RPA foci have disappeared from control cells. One possible consequence of the presence of RPA in RAD51 foci is inhibition of strand invasion and HR. In support of this idea, we observe that *RFWD3*^Δ/Δ^ cells are hypersensitive to the PARP inhibitor olaparib (Figure 5E), which is a hallmark of defective HR (Lord and Ashworth, 2016). This observation is consistent with the previous demonstration of a major defect in HR at a protein-DNA roadblock when cells are depleted of RFWD3 (Elia et al., 2015).

### RPA32 Mutations that Abolish Interaction with RFWD3 Attenuate ICL Repair

**Figure 6:**
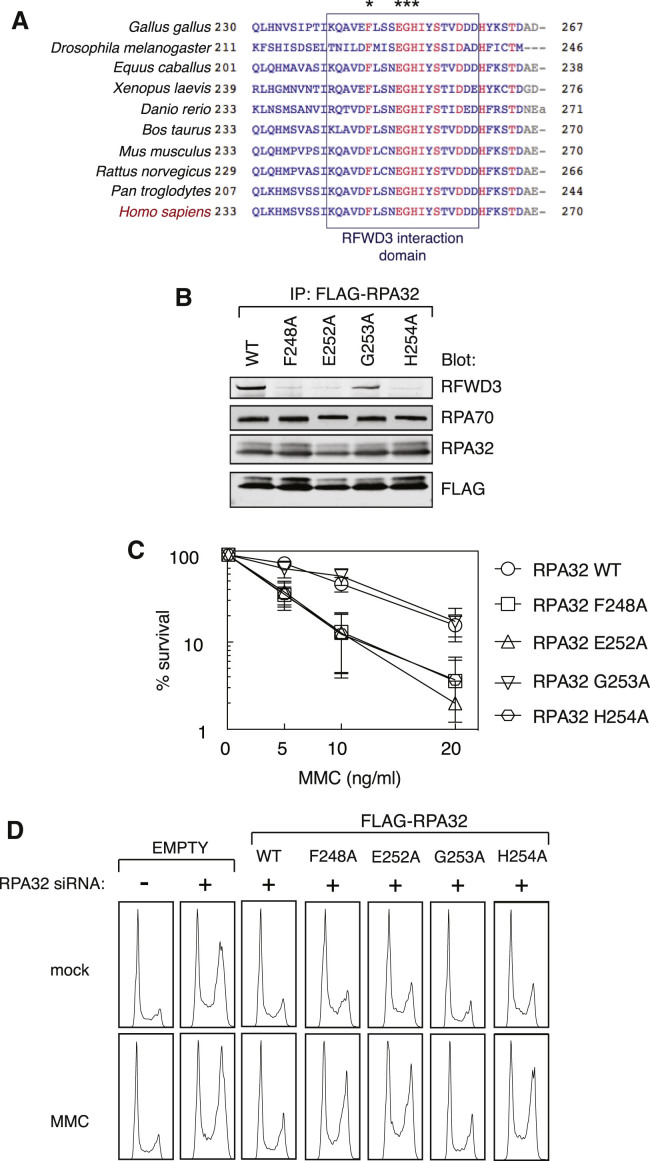
RFWD3 Interaction-Defective RPA Mutants Attenuate ICL Repair (A) Alignment of the C-terminal region of RPA32 orthologs from the indicated species. Conserved residues are highlighted in red. Residues tested by mutational analysis are denoted by asterisks. (B) Anti-FLAG precipitates from extracts of U2OS cells stably expressing FLAG-RPA32 WT or FLAG-RPA32 bearing the indicated mutations were subjected to western blotting with the indicated antibodies. (C) U2OS cells stably expressing FLAG-RPA32 WT or FLAG-RPA32 bearing the indicated mutations were transfected with an siRNA targeting the 3′ UTR of the RPA32 gene. These cells were then subjected to the indicated doses of MMC, and a clonogenic survival assay was carried out. For each cell type, the viability of untreated cells is defined as 100%. Data are represented as mean ± SEM; n = 3. (D) U2OS cells stably expressing FLAG epitope only (EMPTY), FLAG-RPA32 WT, or FLAG-RPA32 bearing the indicated mutations were transfected with an siRNA targeting the 3′ UTR of the RPA32 gene. Cells were then left untreated (mock) or treated with MMC (2ng/mL) for 48 hr before ethanol fixation, propidium iodide staining, and FACS analysis.

Given that RFWD3 affects RPA dynamics at sites of ICL induction and that the FA-associated I639K mutation in RFWD3 abolishes interaction with RPA, we wished to further investigate whether the RPA-RFWD3 interaction is relevant for ICL repair. To this end, we sought to test the effect of mutations in RPA that abolish interaction with RFWD3. It was reported previously that deleting amino acids 243–262 of RPA32 abolishes both the interaction with RFWD3 and RPA ubiquitylation (Elia et al., 2015, Gong and Chen, 2011); however, we sought to make more subtle changes that would disrupt the interaction with RFWD3. As shown in Figure 6A, we identified a number of conserved residues in this region of RPA32, and we tested the effect of mutating four of these residues on the interaction with RFWD3. We found that, although mutating residue G253 to alanine had little effect on the ability of FLAG-tagged RPA32 to interact with endogenous RFWD3, mutating F248, E252, or H254 abolished the interaction of the two proteins (Figure 6B). Importantly, the F248, E252, and H254 mutations did not affect the interaction of RPA32 with RPA70 (Figure 6B).

We next tested the effect of replacing endogenous RPA32 with the mutants that abolish interaction with RFWD3 on ICL repair. To this end, U2OS cells stably expressing FLAG-tagged RPA32 (WT or mutants) were transfected with a small interfering RNA (siRNA) targeting the 3′ UTR of the *RPA32* gene (Figure S7D). The RPA32 siRNA only gave partial depletion of RPA32, which was expected given that complete knockdown of RPA32 is incompatible with cell viability. Strikingly, cells in which RPA32 was replaced by the F248A, E252A, or H254A mutants, which cannot interact with RFWD3, were hypersensitive to MMC, whereas the G253A mutant that can interact with RFWD3 did not render cells any more sensitive to MMC than the WT RPA32 control (Figure 6C). Another hallmark of defective ICL repair is the accumulation of cells in G_2_ phase after MMC exposure. As shown in Figure 6D, control U2OS cells depleted of RPA32 showed a pronounced accumulation in G_2_ phase even without MMC treatment, and this is increased further in response to low doses of MMC. Expression of FLAG-RPA32 (WT) reversed the G_2_ accumulation caused by RPA32 depletion (both with and without exposure of cells to MMC). Interestingly, cells in which endogenous RPA32 was replaced by the F248A, E252A, or H254A mutants, which cannot interact with RFWD3, showed pronounced accumulation in G_2_ only after MMC exposure, indicative of defective ICL repair (Figure 6D). Furthermore, replacing endogenous RPA32 with the G253A mutant, which interacts normally with RFWD3 (Figure 6B), did not cause accumulation of cells in G_2_ after MMC treatment (Figure 6D). These data demonstrate that the interaction of RFWD3 with RPA is required for the optimal repair of ICLs.

## Discussion

In this study, we present evidence that RFWD3 plays an important role in the repair of ICLs. Genome-edited, *RFWD3*-hypomorphic (*RFWD3*^Δ/Δ^) cells show all of the classic hallmarks of defective ICL repair—hypersensitivity to MMC, pronounced G_2_ arrest, and chromosome breakage after MMC exposure (Figure 1). Consistent with this, RFWD3 was one of many factors identified recently in a screen for proteins recruited to chromatin during ICL repair in cell-free *Xenopus* extracts (Räschle et al., 2015). Intriguingly, we were unable to isolate *RFWD3* knockout clones that were completely devoid of RFWD3 protein despite using a range of *RFWD3*-specific gRNAs in multiple cell lines under conditions used to knock out a number of other genes. This suggests that *RFWD3* may be an essential gene, in agreement with the recent identification of *RFWD3* in genome-wide screens for essential genes in KBM7 and HAP1 cells (Wang et al., 2015a). It is unlikely that the essential role of RFWD3 is ICL repair, however, because most FA gene knockouts are viable. On the other hand, several genes controlling HR are essential, including *BRCA1*, *BRCA2*, and *RAD51*. Like *RFWD3*, these genes mediate HR not only during ICL repair but also in response to other DNA lesions and other modes of fork stalling (Deans and West, 2011). Therefore, it is tempting to speculate that the essential function of RFWD3 reflects a critical role in HR at stalled forks. However, the FA-associated I639K mutation completely abolishes interaction with RPA, and yet the patient cells are viable. So if promoting HR is the essential function of RFWD3, then substrates other than RPA must be involved. Further work involving conditional *RFWD3* inactivation and the identification of the full gamut of RFWD3 substrates will be necessary to answer these questions.

We found that the interaction of RFWD3 with RPA appears to be critically important not only for recruitment of RFWD3 to sites of ICL induction but also for ICL repair. We were able to show that replacing endogenous RPA32 with mutants that inhibit the interaction with RFWD3 causes defective ICL repair, whereas a nearby mutation (G253A) that does not affect RFWD3 interaction has no effect on ICL repair (Figure 6). Furthermore, mutating predicted surface hotspots in the RFWD3 WD40 repeats that abolish interaction with RPA also abolishes recruitment to ICLs, whereas WD40 domain mutations that do not affect RPA interaction have no effect on RFWD3 recruitment (Figures 2 and 3). Importantly, the FA-associated I639K mutation completely blocked both RPA binding and RFWD3 recruitment to ICLs induced by psoralen/UV or MMC. Therefore, loss of interaction of RFWD3 with RPA at stalled replisomes is the most likely explanation for the ICL repair defect seen in *RFWD3*-deficient cells and is likely to explain, at least partly, why the I639K mutation causes FA.

We consistently observed that the interaction of RFWD3 with RPA is stimulated by exposure of cells to ICL-inducing drugs. This makes sense from the point of view that RFWD3 is recruited to replication forks only when they stall. However, the mechanism whereby ICL-inducing drugs stimulate the RFWD3-RPA interaction is not clear. RFWD3 is known to be phosphorylated in response to fork stalling, but we found that the ATR inhibitor ETP-46464, which prevents RFWD3 phosphorylation (Figure 4), has no effect on the inducible interaction of RFWD3 with RPA and does not affect RFWD3 recruitment to DNA damage sites (data not shown). Therefore, more work is required to determine how ICL-inducing agents promote the interaction of RFWD3 with RPA.

It is not yet clear whether ubiquitylation of RPA is required for the fork protection and/or ICL repair roles of RFWD3. An RPA32 mutant (K37R K38R) that shows a major reduction in ubiquitylation induced by HU was shown to be proficient for HR and resolution of γH2AX foci, arguing that RPA32 might not be a substrate of RFWD3 relevant for ICL repair (Elia et al., 2015). Although it is possible that it is the ubiquitylation of the other subunits of RPA that is important, currently there is no supporting evidence for this hypothesis. Indeed, it could be argued that RPA simply recruits RFWD3 to stalled forks, where it then ubiquitylates other substrates to promote HR and genome stability. However, one argument in favor of RPA ubiquitylation promoting ICL repair comes from our observation that RPA dynamics at sites of ICL-induced fork stalling are perturbed in *RFWD3*^Δ/Δ^ cells (Figure 5). It is perhaps difficult to explain this defect without invoking RFWD3 catalytic activity, and, on this basis, we speculate that RPA ubiquitylation facilitates the unloading of RPA from resected DNA ends during HR. It is not yet clear how RFWD3-catalyzed ubiquitylation might facilitate RPA dissociation. It may be that RFWD3 increases the turnover of RPA to promote dissociation from broken replication forks, and, in this light, it is worth noting that inactivating RFWD3 catalytic activity boosts the levels of this protein, suggesting that it might control its own turnover (Figures 2A and S5). It is also possible that RPA ubiquitylation enhances its interaction with one or more proteins that facilitate its extraction from ssDNA, such as the p97 segregase. Testing these possibilities will be an interesting line of future investigation.

Even if RPA ubiquitylation by RFWD3 is required for RPA unloading from ICL-induced foci, it does not necessarily follow that the persistence of RPA seen in *RFWD3*^Δ/Δ^ cells is sufficient to derail the HR process, especially because RAD51 appears to load normally in these cells. In other words, altered RPA dynamics may not be the cause of the HR defects noted in *RFWD3*-deficient cells (Figure 5E; Elia et al., 2015). However, it is possible that the presence of RPA in RAD51 foci impedes the formation of the RAD51 nucleofilament, thereby inhibiting strand invasion and HR. It therefore seems reasonable to speculate that a failure in HR—caused perhaps by loss of RPA ubiquitylation—is responsible for both the defect in ICL repair and the FA disease phenotype seen in the context of RFWD3 mutations. This is particularly relevant given that mutations in key HR factors such as BRCA1 and BRCA2 have also been found to cause major defects in ICL repair as well as FA (Deans and West, 2011).

Over the past decade it has become clear that the replacement of RPA with RAD51 on resected forks is a complex transaction requiring a range of proteins, including the mediator proteins RAD51B–RAD51D, XRCC2, and XRCC3 (Krejci et al., 2012). The BRCA2 tumor suppressor is also involved in this process, promoting the assembly of a RAD51 nucleofilament on ssDNA both in the context of ICL repair as well as the repair of DSBs in general (Krejci et al., 2012, Liu et al., 2010). Additionally, other proteins, such as the MMS22L-TONSL complex, have been implicated in replacing RPA with RAD51, at least in the context of replication-associated DSBs (Duro et al., 2010, O’Donnell et al., 2010). Determining exactly how the removal of RPA is coordinated with RAD51 loading and nucleofilament formation will be a major challenge for the future, as will determining the precise role of RFWD3 in this surprisingly complex process.

## STAR★Methods

### Key Resources Table

**Table:** 

REAGENT or RESOURCE	SOURCE	IDENTIFIER
**Antibodies**		

RFWD3	Abcam	ab138030
RPA32 (immunoblot)	Bethyl	A300-244A; RRID: AB_185548
RPA32 (immunofluorescence)	Abcam	ab2175; RRID: AB_302873
RPA70	Abcam	ab79398; RRID: AB_1603759
BRCA1 phospho-Ser1457	Bethyl	A300-009A; RRID: AB_155882
GFP (immunoblot)	DSTT, University of Dundee	S268B
GFP (immunofluorescence)	Abcam	ab13970; RRID: AB_300798
ERCC1	DSTT, University of Dundee	S185D
FLAG	Cell Signaling	2368
phospho-H2A.X	Cell Signaling	2577; RRID: AB_2118010
RAD51	Bioacademia	70-001
FANCD2	DSTT, University of Dundee	S099D
GAPDH	Cell Signaling	2118; RRID: AB_561053

**Chemicals, Peptides, and Recombinant Proteins**		

MBP-RFWD3	this paper	N/A
ETP46464 (ATR inhibitor)	Toledo et al., 2011	N/A
KU59933 (ATM inhibitor)	Hickson et al., 2004	N/A

**Critical Commercial Assays**		

CellTiter 96 cell proliferation assay kit (MTS assay)		G5421
Duolink Probemaker Plus kit		DUO92009
Duolink Probemaker Minus kit		DUO92010
Duolink detection kit green		DUO92014

**Deposited Data**		

Raw imaging data	this paper	Mendeley data: http://dx.doi.org/10.17632/brzwywcrbj.1

**Experimental Models: Cell Lines**		

U2OS FRT	in house	N/A
HeLa FRT FLAG-CAS9	Adrian Saurin lab	N/A
RFWD3 hypomorphic HeLa cells	this paper	N/A
ERCC1 knockout HeLa cells	this paper	N/A

**Oligonucleotides**		

Scramble siRNA: UCCACAACAUAAUCCUAAUUU	Invitrogen	N/A
FANCD2 siRNA #1: CAGAGUUUGCUUCACUCUCUAUU	Invitrogen	N/A
FANCD2 siRNA #2: CGGCUUCUCGGAAGUAAUUUAUU	Invitrogen	N/A
RPA32 siRNA: CCUAGUUUCACAAUCUGUU	Invitrogen	N/A
RFWD3 gRNA cloning forward primer: GGAAAGGACGAAACACCGAAGTTACATGTATCGATGGGTTTTAGAGCTAGAAAT	Invitrogen	N/A
RFWD3 gRNA cloning reverse primer: ATTTCTAGCTCTAAAACCCATCGATACATGTAACTTCGGTGTTTCGTCCTTTCC	Invitrogen	N/A
RFWD3 sequencing forward primer: TGGTACCTGGGGAAGTTGTC	Invitrogen	N/A
RFWD3 sequencing reverse primer: CTTAAACACGGGAGGCAGAG	Invitrogen	N/A
ERCC1 cloning forward primer: GGAAAGGACGAAACACCGATACCCCTCGACGAGGATGGTTTTAGAGCTAGAAAT	Invitrogen	N/A
ERCC1 cloning reverse primer: ATTTCTAGCTCTAAAACCATCCTCGTCGAGGGGTATCGGTGTTTCGTCCTTTCC	Invitrogen	N/A
ERCC1 sequencing forward primer: GGAGCTCTCGGAGTTTTGTG	Invitrogen	N/A
ERCC1 sequencing reverse primer: AGTGTCTGGCCTCTGCCTTA	Invitrogen	N/A

**Recombinant DNA**		

pcDNA5 FRT/TO GFP-RFWD3	this paper	DU49512
pcDNA5 FRT/TO RFWD3-GFP	this paper	DU49591
pcDNA5 FRT/TO GFP-RFWD3 C315A	this paper	DU49582
pcDNA5 FRT/TO GFP-RFWD3 I639K	this paper	DU49590
pcDNA5 FRT/TO GFP-RFWD3 C315A I639K	this paper	DU50397
pcDNA5 FRT/TO GFP-RFWD3 C315A W543A	this paper	DU53212
pcDNA5 FRT/TO GFP-RFWD3 C315A Q499A	this paper	DU53211
pcDNA5 FRT/TO GFP-RFWD3 C315A L518A	this paper	DU53189
pcDNA5 FRT/TO 6xHis-3xFLAG-RPA32	this paper	DU53226
pcDNA5 FRT/TO 6xHis-3xFLAG-RPA32 F248A	this paper	DU53308
pcDNA5 FRT/TO 6xHis-3xFLAG-RPA32 E252A	this paper	DU53309
pcDNA5 FRT/TO 6xHis-3xFLAG-RPA32 G253A	this paper	DU53310
pcDNA5 FRT/TO 6xHis-3xFLAG-RPA32 H254A	this paper	DU53315

### Contact for Reagent and Resource Sharing

Further information and requests for resources and reagents should be directed to and will be fulfilled by the Lead Contact, John Rouse (j.rouse@dundee.ac.uk).

### Experimental Model and Subject Details

#### Cell lines and tissue culture

All cell lines used in this study were derived from U2OS FRT Flp-In TRex or HeLa FRT FLAG-Cas9 cells. These cell lines were grown in Dulbecco’s Modified Eagle Medium (DMEM) plus 10% fetal bovine serum (FBS), 1% penicillin/streptomycin and 1% L-glutamine. Additionally, 100 μg/ml hygromycin and 15 μg/ml blasticidin were added for the culture of Flp-In TRex stable cell lines. All cell lines were grown at 37°C and 5% CO_2_.

### Method Details

#### Genome editing

Genome editing was carried out as described previously (Munoz et al., 2014). Briefly, Cas9 expression in HeLa FRT cells stably expressing FLAG-tagged Cas9 was induced by addition of tetracycline (1 μg/ml) and after a further 16h cells were transfected with a plasmid expressing a guide (g)RNA targeting either exon 4 of *RFWD3* (GAAGTTACATGTATCGATGGAGG) or exon 2 of *ERCC1* (GATACCCCTCGACGAGGATGAGG). Cells were then selected with puromycin (3 μg/ml) for 24h, before single cell cloning. Individual clones were grown up, and screened for knockout by western blot and PCR followed by restriction enzyme digest with ClaI (RFWD3) or Taq1 (ERCC1).

#### RT-PCR and sequencing

RT-PCR was carried out as previously described (Lachaud et al., 2016a). Briefly, total mRNA was extracted from cells using a QIAGEN RNeasy kit and RT-PCR carried out using a Clontech RT-PCR kit. Oligos were designed to amplify sequences between exons 3 (TGAGAGCACCATTGGATGC) and 5 (ACAGCGTAATGCTGAGAGCC) of the RFWD3 mRNA. Sequencing was carried out by the DNA sequencing team at the University of Dundee.

#### Cell lysis and immunoprecipitation

Cells were lysed in ice cold buffer: 50mM Tris-HCl pH 7.5 plus 150mM NaCl, 270mM sucrose, 1% Triton X-100 and 0.5% NP40 with protease inhibitor cocktail (Roche), 10mM iodoacetamide, 10ng/ml microcystin-LR and 0.5U/ml benzonase. Lysates were incubated on ice for 30 min. All immunoprecipitations were carried out at 4°C for 2h, except for FLAG-RPA32 immunoprecipitations that were carried out for 16 hr.

#### MTS assay

Cells were diluted to 2x10^4^ cells/ml and 100 μL of cell suspension was seeded per well in a 96 well plate. After 24h, cells were treated with MMC (100 ng/ml) and incubated for a further 48h. Finally, 20 μL MTS reagent was added per well, and absorbance at 490nm was read. For each cell line, the viability of untreated cells was taken to represent a cell viability of 100%.

#### Clonogenic survival assay

For HeLa cells, 750 cells were seeded per 10cm dish and allowed to adhere for 4-6h. For U2OS, 1000 cells were seeded per 10cm dish and allowed to adhere for the same time. Cells were then treated with the relevant genotoxin for 16h (for MMC, cisplatin and HU), at which point cells were washed free of drugs. In the case of IR, no media change was required. Likewise, no media change was carried out for olaparib as chronic treatment is required. Cells were then incubated for 1 – 2 weeks until visible colonies had formed. Colonies were washed with 1x PBS followed by methanol, then stained with crystal violet, washed with water and counted.

#### Cell cycle analysis

Cells were seeded at low confluency in 10cm dishes and allowed to adhere before being treated with 2ng/ml MMC for 24 hr (HeLa) or 48 hr (U2OS). Cells were then fixed in 70% ethanol and stained with propidium iodide. Cell cycle distribution was analyzed using a FACS Canto machine.

#### Chromosome abnormalities

Chromosome abnormalities were measured as previously described (Castor et al., 2013). Briefly, cells were seeded at low confluency in 10cm dishes, and allowed to adhere before being treated with 2ng/ml MMC. After 24 hr, cells were treated with 0.1 μg/ml colcemid (Sigma) for 2 hr. Samples were then harvested, washed with PBS, and incubated with 75mM KCl for 20 min at 37°C. Samples were fixed in methanol/acetic acid (3:1) and dropped on slides to generate metaphase spreads. Chromosomes were stained with Giemsa (VWR) and abnormalities were counted using a 100x objective on a Zeiss 710 confocal microscope.

#### Psoralen/UV laser micro-irradiation

Localized micro-irradiation was induced as previously described (Perez-Oliva et al., 2015). Briefly, cells were seeded in glass-bottomed dishes and treated with 1 μg/ml tetracycline as required to induce GFP-RFWD3 expression. After 24h, cells were incubated with psoralen (50 μM) in Leibowitz medium for 2h. A Zeiss PALM microdissection microscope equipped with a 360nm UV laser was then used to damage 100 nuclei per dish.

#### Immunofluorescence and confocal microscopy

Cells were pre-extracted with 0.2% Triton X-100 for 2-5 min, then fixed for 10 min in 3% paraformaldehyde. Permeablization was carried out for 20 min with 0.2% Triton X-100, followed by blocking for 1h at room temperature in antibody dilution buffer (PBS plus 5% donkey serum, 0.1% fish skin gelatin, 0.1% Triton X-100, 0.05% Tween and 0.05% sodium azide). Samples were then incubated in primary antibody overnight at 4°C, washed with 0.2% PBS-Tween, and incubated with secondary antibody for one hour at room temperature in the dark. Finally, samples were washed with 0.2% PBS-Tween, DAPI stained and imaged using a Zeiss 710 confocal microscope.

#### High resolution microscopy

Samples were prepared as per the standard immunofluorescence protocol (see above), then mounted with coverslips using Prolong Diamond anti-fade mountant (Thermo Fisher). High-resolution images of RPA and RAD51 foci in single nuclei were then obtained with Hyvolution software on a Leica SP8 confocal microscope, using a 63x oil immersion lens and a pinhole of 0.5 AU.

#### In vitro ubiquitylation assay

1 μg RFWD3 (wild-type or C315A) was incubated in reaction buffer (250mM HEPES, 10mM MgCl_2_, 0.5mM TCEP) with 100ng E1, 200ng UBC13-UEV1A and 2 μg ubiquitin at 30°C. Reactions were started with the addition of 0.5mM ATP. Reactions were stopped after 30 min by the addition of loading buffer (Nupage) supplemented with reducing agent.

#### Proximity ligation assay (PLA)

Cells were pre-extracted, fixed, stained and blocked as per the standard immunofluorescence procedure (see above). Primary antibodies conjugated to either Duolink Plus or Duolink Minus probes were generated using a Sigma Duolink probemaker kit as per manufacturer’s instructions. Samples were incubated with conjugated primary antibodies overnight at 4°C, then washed with 1x wash buffer A (10mM Tris-HCl pH7.5, 150mM NaCl, 0.05% Tween), and incubated with ligase diluted 1:40 in 1x ligation buffer (Sigma Duolink detection kit green) for 30 min at 37°C. Next, samples were washed again with 1x wash buffer A then incubated for 100 min at 37°C with polymerase diluted 1:80 in 1x amplification buffer (Sigma Duolink detection kit green). Finally, samples were washed with 1x wash buffer B (200mM Tris-HCl pH7.5, 100mM NaCl) followed by 0.01x wash buffer B, then DAPI stained and imaged using a Zeiss 710 confocal microscope.

### Quantification and Statistical Analysis

Details of statistical analysis can be found in the legends to the relevant figures. In each case, the sample size (n) refers to the number of independent experiments, and data are represented as mean ± standard error of the mean (SEM).

### Data and Software Availability

The raw data files for imaging data presented in this manuscript have been deposited to Mendeley Data and are available at http://dx.doi.org/10.17632/brzwywcrbj.1.

## Author Contributions

Conceptualization, J.R. and D.S.; Methodology, J.R., L.F., I.M.M., and C.L.; Investigation, L.F., I.M.M., and C.L.; Resources, R.T. and P.L.A.; Writing – Original Draft, J.R. and L.F.; Writing – Review & Editing, J.R. and L.F.; Funding Acquisition, J.R.; Supervision, J.R.

## Supplemental Information

Document S1. Figures S1–S7

Document S2. Article plus Supplemental Information
