# Supplementary material for: RPA-Mediated Recruitment of the E3 Ligase RFWD3 Is Vital for Interstrand Crosslink Repair and Human Health
Source: Mol Cell. 2017 Jun 1;66(5):610–621.e4. doi: 10.1016/j.molcel.2017.04.021 (PMC5459755; doi:10.1016/j.molcel.2017.04.021)
Supplement: Document S1. Figures S1–S7 [file mmc1.pdf]

Molecular Cell, Volume *66*

Supplemental Information

RPA-Mediated Recruitment of the E3 Ligase RFWD3 Is Vital for Interstrand Crosslink Repair and Human Health

Laura Feeney, Ivan M. Muñoz, Christophe Lachaud, Rachel Toth, Paul L. Appleton, Detlev Schindler, and John Rouse

Supplemental Information Inventory

Supplemental Data

Fig. S1 Model for replication-dependent ICL repair; related to Fig. 1

Fig. S2 Generating RFWD3-hypomorphic human cells; related to Fig. 1

Fig. S3 Analysis of hypersensitivity of *RFWD3*$^{\Delta/\Delta}$ cells to ICL-inducing agents; related to Fig. 1

Fig. S4 Compound heterozygous *RFWD3* mutations in a new FA subtype; related to Fig. 2

Fig. S5 RFWD3 C315A mutant is inactive; related to Fig. 2

Fig. S6 RFWD3 co-localizes with RPA at ICLs; related to Fig. 3

Fig. S7 RFWD3 affects RPA dynamics; related to Fig. 5 and Fig. 6

Feeney_Fig_S1

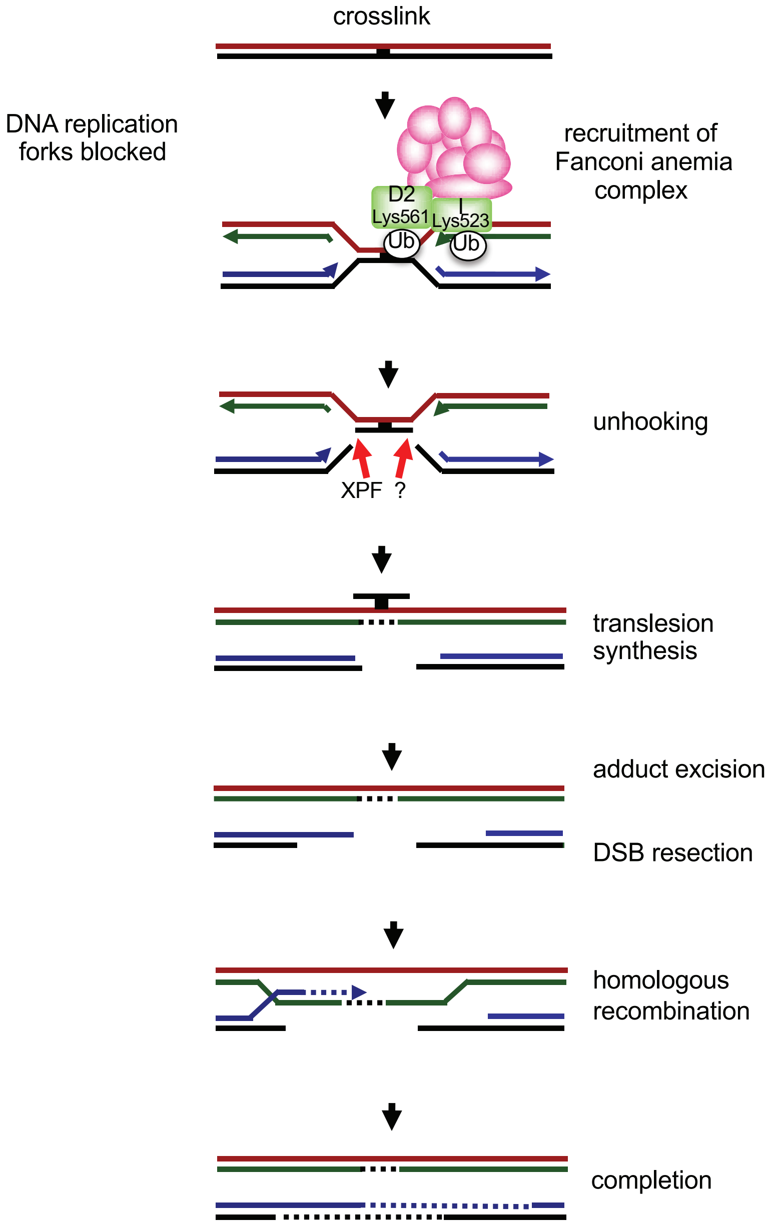

Fig. S1 Model for replication-dependent ICL repair (related to Fig. 1)

Evidence exists for different modes of ICL repair in S-phase. In one of these models, it has been proposed that ICL repair is initiated by the convergence of two replication forks on the ICL. Various signaling proteins including the Fanconi Anemia (FA) core complex are recruited to the vicinity of the blocked replisome. This triggers the mono–ubiquitylation of FANCD2 and its paralog FANCI at Lys561 and Lys523 respectively, that in turn directs subsequent steps of ICL repair. Cleavage of the leading strand template of one of the forks in concert with cleavage of the same strand on the opposite side of the ICL would unhook the ICL. This results in two one–ended DSBs and a gapped duplex with the ICL adduct on one strand that is filled in by translesion synthesis. The ICL adduct is excised and resection of one of the DSBs generated by unhooking initiates homologous recombination that completes repair.

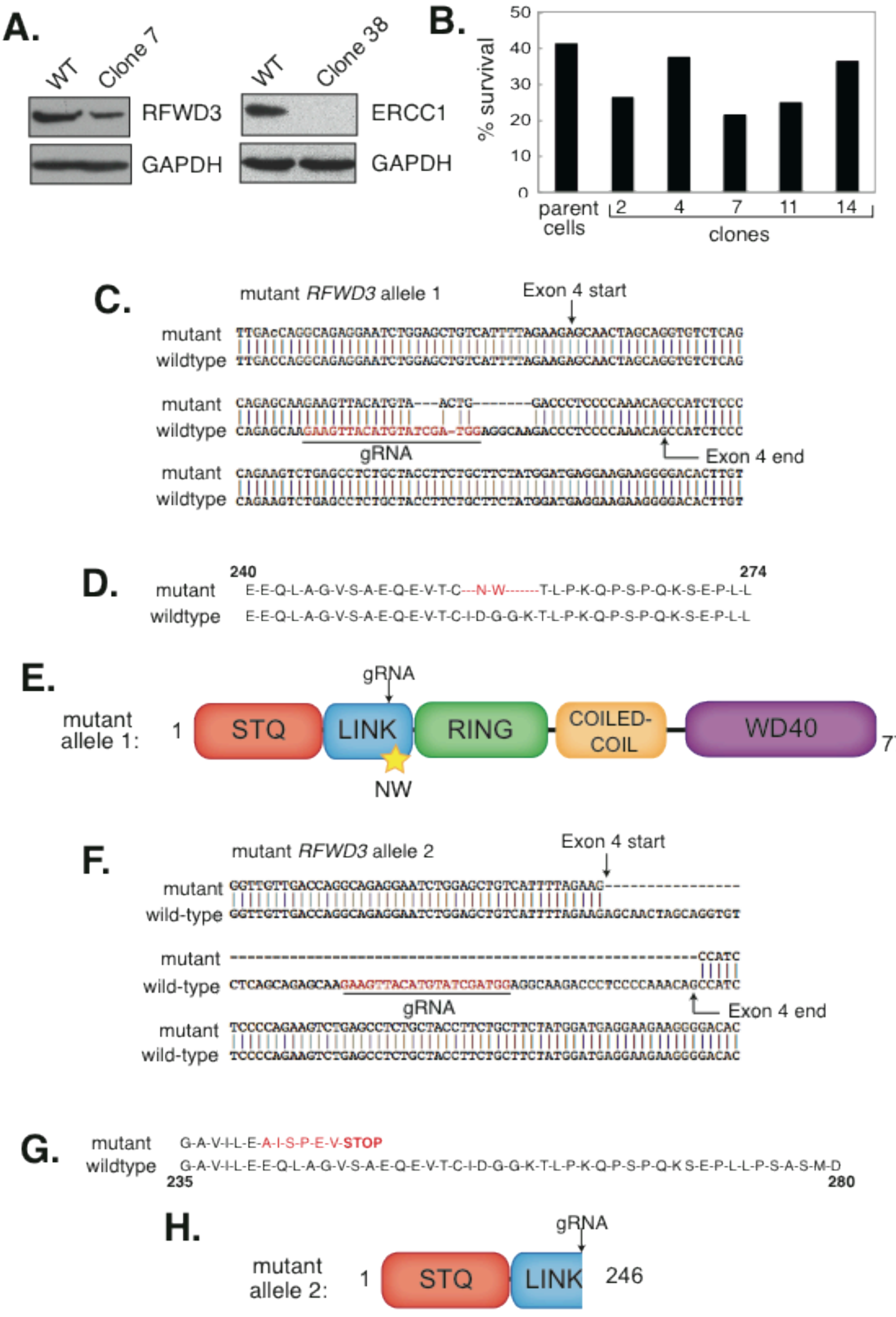

Feeney_Fig_S2

Fig. S2. Generation of RFWD3-hypomorphic human cells (related to Fig. 1)

(A) Extracts from cells of the genotypes indicated were subjected to western blotting with the antibodies indicated.

(B) Cells from puromycin-resistant clones obtained after genome editing to disrupt *RFWD3* using a gRNA directed to exon 4 were seeded in 96 well plates. Cells were either left untreated, or treated with MMC. After 48 hours, MTS reagent was added and absorbance at 490nm read. Viability of untreated cells was defined as 100%. Only data from cells exposed to MMC is shown.

(C) The sequence of the first mutant allele in *RFWD3*$^{\Delta/\Delta}$ HeLa cells (clone 7) is aligned with the sequence of *RFWD3* from control cells. The binding site of the exon 4-targeting gRNA is highlighted in red. The start and end points of exon 4 are indicated by arrows.

(D) The predicted protein sequence in the region of RFWD3 affected by the mutation in the first mutant allele in *RFWD3*$^{\Delta/\Delta}$ HeLa cells in (B). The alteration in amino acid sequence is highlighted in red.

(E) Schematic of the human RFWD3 protein, showing the predicted effect of the first mutant allele in compound heterozygous *RFWD3*$^{\Delta/\Delta}$ HeLa cells (yellow star). Modular domains are color-coded. "STQ" indicate a Ser/Thr-rich region containing multiple consensus sites for phosphorylation by the ATM/ATR kinases. The region of RWFD3 encoded by the sequence in exon 4 targeted by the gRNA used to generate *RFWD3*$^{\Delta/\Delta}$ cells is shown with an arrow.

(F-H) Same as (C-E) except that they refer to the second mutated allele in compound heterozygous *RFWD3*$^{\Delta/\Delta}$ HeLa cells (clone 7).

Feeney_Fig_S3

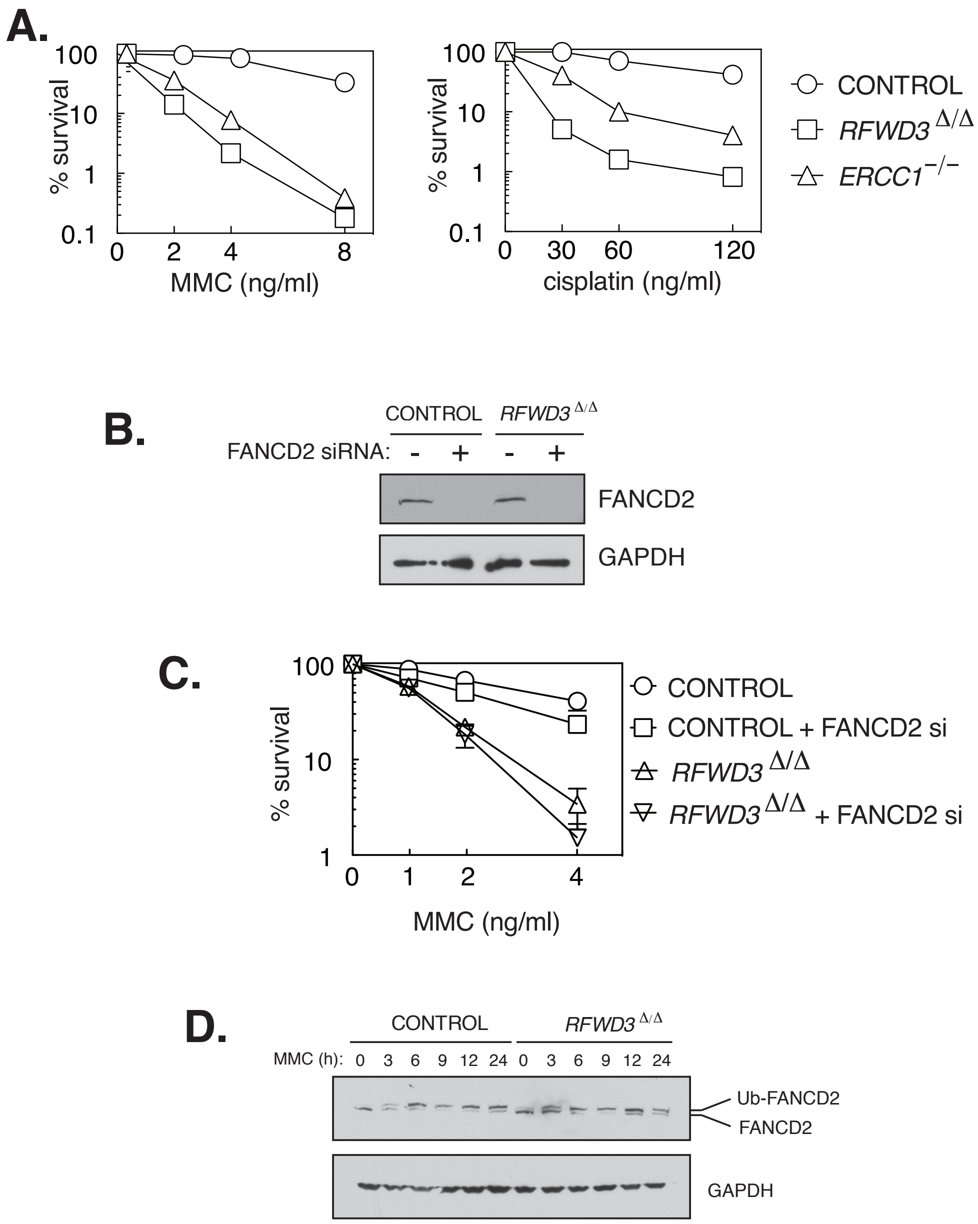

Fig. S3 Analysis of hypersensitivity of RFWD3$^{Δ/Δ}$ cells to ICL-inducing agents (related to Fig. 1)

(A) Clonogenic survival analysis of control, *RFWD3*$^{Δ/Δ}$ and *ERCC1*$^{-/-}$ HeLa cells exposed to the doses of MMC (left panel) or cisplatin (right panel) indicated. For each cell type, viability of untreated cells is defined as 100%. Data are represented as mean ± SEM, *n*=3.

(B) *RFWD3*$^{Δ/Δ}$ HeLa cells or parental cells treated were transfected with FANCD2-specific siRNA or a scrambled siRNA. Cells were lysed and subjected to western blotting with the antibodies indicated.

(C) Cells from (B) were subjected to clonogenic survival analysis with MMC. For each cell type, viability of untreated cells is defined as 100%. Data are represented as mean ± SEM, *n*=3.

(D) *RFWD3*$^{Δ/Δ}$ HeLa cells or parental cells treated were exposed to MMC for the times indicated and extracts were subjected to western blotting with FANCD2 antibodies to monitor FANCD2 ubiquitylation.

Feeney_Fig_S4

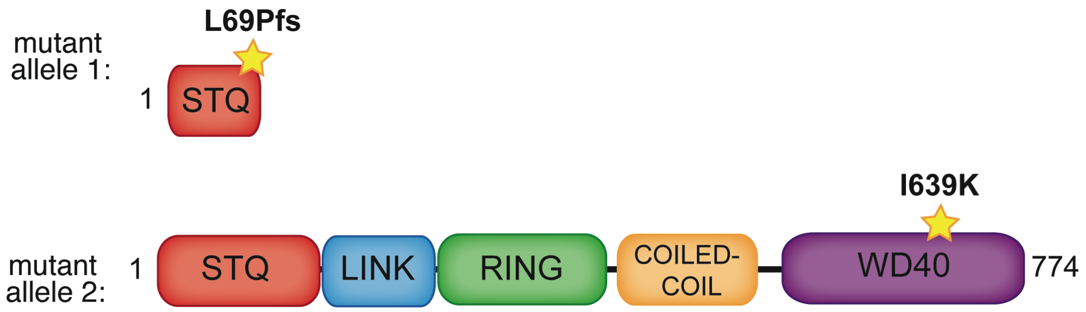

Fig. S4 Compound heterozygous *RFWD3* mutations in a new FA subtype (related to Fig. 2)

Schematic of the human RFWD3 protein, with yellow stars indicating the mutations found in the new FA subtype alluded to in the manuscript (DS; manuscript in revision). Modular domains are color-coded. “STQ” indicates a Ser/Thr-rich region containing multiple consensus sites for phosphorylation by the ATM/ATR kinases.

Feeney_Fig_S5

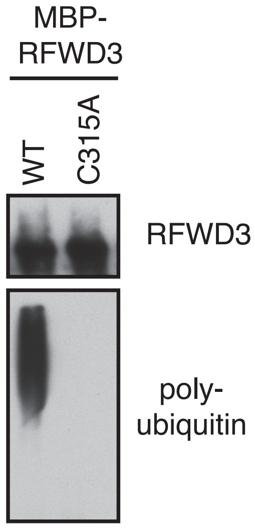

Fig. S5 The RFWD3 C315A mutant is inactive (related to Fig. 2)
RFWD3 (wild type or C315A) was incubated with E1, UBC13-UEV1A, ubiquitin and ATP. Reactions were stopped after 30 min, and western blot analysis was carried out with the indicated antibodies.

Feeney_Fig_S6

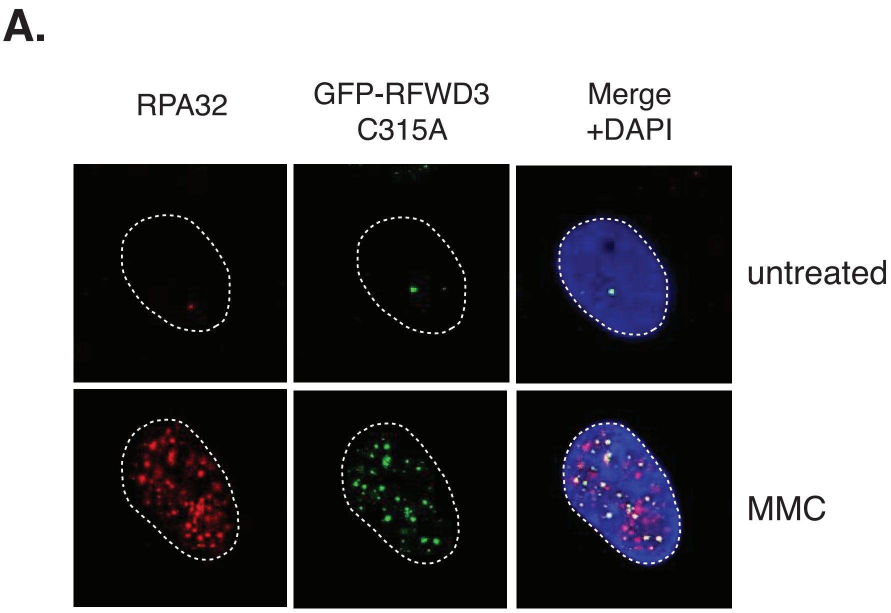

Fig. S6 RFWD3 co-localizes with RPA at ICLs (related to Fig. 3)

U2OS cells stably expressing GFP-RFWD3 were incubated with or without MMC for 16h. Cells were fixed and subjected to indirect immunofluorescence analysis with antibodies against RPA32 or GFP.

Feeney_Fig_S7

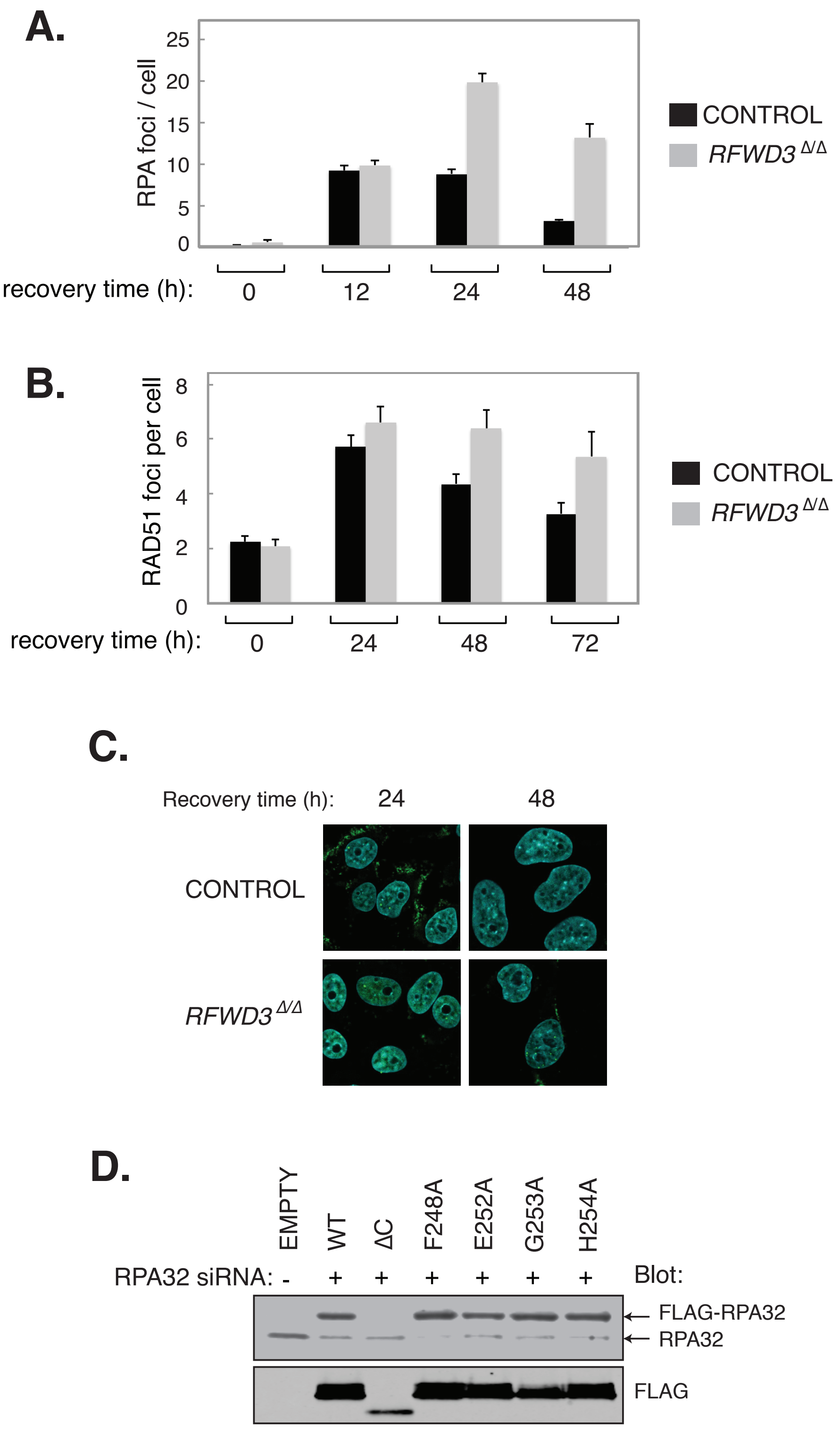

Fig. S7 RFWD3 affects RPA dynamics (related to Fig. 5 and Fig. 6)

(A, B) Control or *RFWD3*$^{\Delta/\Delta}$ HeLa cells were exposed to MMC for 2h, then washed free of drug and allowed to recover for the times indicated. Cells were pre-extracted, fixed and subject to immunofluorescence analysis to measure the number of RPA (A) or RAD51 (B) foci per cell in a field of 100 cells. Control cells are parental cells that were taken through genome editing protocols but are wild type for RFWD3. Data are represented as mean ± SEM, n=3.

(C) Control or *RFWD3*$^{\Delta/\Delta}$ HeLa cells were exposed to MMC for 2h, then washed free of drug and allowed to recover for the times indicated. Cells were pre-extracted, fixed and incubated with RPA32 and RAD51 primary antibodies conjugated to oligonucleotides. Ligation and amplification with fluorescent dNTPs were then carried out, followed by DAPI staining. Samples were then imaged on a Zeiss 710 confocal. Representative images of the foci observed are shown for each time point.

(D) U2OS cells stably expressing FLAG epitope only (EMPTY), FLAG-RPA32 wild type (WT), or FLAG-RPA32 bearing the mutations indicated were transfected with an siRNA targeting the 3’UTR of the RPA32 gene. “ΔC” refers to an RPA32 deletion mutant truncated from amino acid 242. Cell extracts were subjected to western blotting with the antibodies indicated.
